# “They Were Really Looking for a Male Leader for the Building”: Gender, Identity and Leadership Development in a Principal Preparation Program

**DOI:** 10.3389/fpsyg.2016.00141

**Published:** 2016-02-16

**Authors:** Laura J. Burton, Jennie M. Weiner

**Affiliations:** Department of Educational Leadership, Neag School of Education, University of ConnecticutStorrs, CT, USA

**Keywords:** leadership development, education, gender stereotypes, bias, principal preparation

## Abstract

This study utilized a comparative case study analysis to investigate how gender influenced the experiences of participants in a leadership development program (principal preparation program) designed to lead public K-12 schools identified as requiring turnaround. We closely focused on two participants, a man and a woman, and compared the ways each participant made meaning of his/her experiences as developing leaders in the program. Although both participants conceptualized effective leadership in similar communally-oriented ways, the way they came to construct their identities as leaders varied greatly. These differences were largely influenced by different and, what appeared to be, gendered feedback occurring during the program and when participants entered the job market.

## Introduction

Public school teaching (K-12) is largely a female dominated profession, with more than 86% of teachers in the United States being women (Feistritzer et al., 2011; Grissom et al., 2013); however, as true in many industries, few women move up the ranks to serve as institutional leaders. In the U.S. for example, women comprise only 52% of K-8 principals and 30% of high school principals. One reason for these lower numbers is that women are also disproportionately underrepresented in principal preparation programs suggesting pipeline issues may be as much to blame as a lack of longevity in the field (Darling-Hammond et al., 2007). And yet, research regarding the underrepresentation of women principals has often focused on considering the barriers and supports, exclusive of preparation, female principals face when becoming and working as school leaders. Moreover, the scope of the research is often narrow and has yet to probe how gender identity and role may influence preparation program participants' understandings of who should be a principal or how the role should be enacted (see Sperandio and LaPier, 2009, as an exception). When such studies do exist, they tend to focus singularly on women's experiences rather than investigate how the gendered construction of leadership impacts all participants whether they be female or male. Further, there is little research available that examines the needs of women within the context of leadership development programs (Harris and Leberman, 2012), and to our knowledge only one study that directly explores how gender biases and stereotypes must be addressed to best support women's leadership development (Ely et al., 2011) thus making it an issue in need of further exploration.

Social role theory gives insights into how women and men are differently evaluated for positions of leadership or other higher status positions and therefore provides a useful framework to consider how leadership and the process of leadership development occurs within the context of principal preparation programs. Both role congruity theory (Eagly and Karau, 2002) and status incongruity theory (Rudman et al., 2012) utilize concepts of social role theory to provide a means to recognize the challenges women face when seeking to lead or leading in an organizational setting (e.g., the principalship). Social role theory (Eagly and Wood, 2012) proposes that there are differing expectations regarding behaviors and attributes considered appropriate to men and women (i.e., social role stereotypes). For women, demonstrating nurturing, caring, and demur behaviors are both desirable (prescribed) and expected (descriptive). These behaviors and attributes are described as communal. Conversely, men are expected and desired to behave in more agentic ways (e.g., aggressive, dominant, self-confident) (Eagly and Wood, 2012). As described in role congruity theory (Eagly and Karau, 2002), women face a double bind in the context of leadership, as prejudice occurs when there is a perceived incongruity between group stereotypes (e.g., leadership) and social role stereotypes (Koenig and Eagly, 2014). Further, status incongruity theory (Rudman et al., 2012) notes that this double bind serves to reinforce a gendered hierarchy in positions of leadership, as those holding stronger beliefs about gender norm stereotypes provide harsher evaluations of women in leadership positions.

Importantly, we recognize that gender is only one of multiple identities individuals hold (e.g., race, ethnicity, sexuality), and that those identities intersect to influence each person's experiences (Rusch, 2004). However, even with this acknowledgement of people's multiple identities and the intersectionality across and between them and leadership (Collins, 1998), when race is considered, gender bias against women remains significant (Reed, 2012) and has been shown to exist within and across many racial boundaries (white males/black women, black men/black women, etc.) (Brooks and Jean-Marie, 2007). In this way, women of color who inhabit, or attempt to inhabit, leadership positions are subject to the “double jeopardy” of potential discrimination, both for their gender and their race (Rosette and Livingston, 2012). The saliency of gender bias has surfaced in previous researcher that examined experience of women in a leadership development program. These women had varied backgrounds along multiple dimensions and findings suggested a need for deeper investigation, specifically in the area of gender, when examining women's experiences in leadership development programs (Weiner and Burton, unpublished paper).

Therefore, utilizing social role theory as our theoretical framework, we contend that stereotyping a principal as a masculine role (i.e., suggesting it requires agentic characteristics) shapes the construction of leadership development within principal preparation programs and subsequently the experiences of men and women enrolled in them. This is important because, if, within such programs, the construction of what it means to be a principal is gendered based on stereotypes, and evaluation of being “effective” is also derived based on these prescribed social roles, women participants may experience the challenges described by role congruity theory (Eagly and Karau, 2002) and the status incongruity hypothesis (Rudman et al., 2012). In particular, women participants in principal preparation programs may be perceived as lacking the necessary skills to be principals and will be differently evaluated internally in the program, as they seek out positions as principals and later as they inhabit the role.

### Women in educational leadership

A growing area of interest among researchers is better understanding the persistent and pernicious gender leadership gap in education (e.g., Reed, 2012; Nichols and Nichols, 2014). Work by Nichols and Nichols (2014) and others (e.g., Tallerico, 2000; Stufft and Coyne, 2009; Grogan and Shakeshaft, 2011) suggests that despite a need to investigate issues of diversity and school leadership more broadly, including the intersection of race, sexuality and gender, gender itself is both a valid way to consider leadership and one which requires additional study (Rusch, 2004; Coleman and Fitzgerald, 2008). Taking up this charge, and acknowledging that these other identity issues concurrently influence aspirant principals' experiences, in the following section, we provide a historical perspective of the intersection of gender and school leadership to help contextualize the study and our findings.

Teaching has long been considered to be a “feminized” profession (e.g., Apple, 1985; Carrington and McPhee, 2008; Goldstein, 2014), fitting neatly with traditional societal expectations regarding women's role as caretakers (Strober and Tyack, 1980; Grumet, 1988). Indeed, teaching was often seen as a natural extension of these expectations with female teachers serving as nurturers of children's minds (Weiler, 1989; Nelson, 1992). Over time, society and female teachers themselves came to normalize and internalize these views—focusing their energies on building caring and close relationships with students and treating administration as a male endeavor (Adams and Hambright, 2004). Given this history of the profession it is not surprising that even today many female teachers refrain from becoming administrators or feel a sense of role conflict if they do (Loder and Spillane, 2005).

Frames placing women in the classroom and men in the principal's office were and continue to be reinforced in schools with women often being actively discouraged or disallowed from taking on administrative roles (Strober and Tyack, 1980; Sanchez and Thornton, 2010). Like many male dominated professions, school leadership is often described as an “old boys club” with males receiving formal and informal mentoring to succeed and women receiving fewer supports (Peters, 2010; Muñoz et al., 2014). As Gardiner et al. (2000) and others have highlighted (e.g., Kanter, 1993), such cultural norms tend to self-perpetuate as those most often in positions of authority (i.e., white males) provide professional support and guidance to others most like themselves. Even today, white males continue to be a minority among teachers but a majority in administration having been identified as “having leadership potential” (Cognard-Black, 2004; Myung et al., 2011) and quickly moved up the ranks (i.e., riding the “glass escalator”) (Williams, 1992). Moreover, emergent research suggests that gender bias cuts across other dimensions of identity, and race in particular, with females of color facing discrimination in both arenas, thus limiting their access to leadership relative to male colleagues (Banks, 2000; Reed, 2012). In this way, gender appears to be a salient feature across all types of emergent leaders.

At the same time men often receive greater support to become principals, their dominance in the position may also be perpetuated and reinforced by discourses emphasizing more agentic or stereotypical masculine modes of leadership (Gill and Arnold, 2015). Indeed, despite a move in recent years for schools to introduce more shared or distributed leadership models (Elmore, 2000; Spillane et al., 2001; Supovitz and Tognatta, 2013), educators often construct the principal position as that of a “lone hero” (Higgins et al., 2009). Such models push the principal to be “strong, decisive and in charge” (Yep and Chrispeels, 2004, p. 164) and that they take a more “directive approach” in their practice (Lezotte, 1994; Leithwood et al., 2010). Moreover, many have argued that this orientation toward principals enacting strong, autocratic and aggressive leadership has been exacerbated by recent education policies emphasizing accountability (Hamilton et al., 2007) and is particularly true when a school is deemed underperforming (Shiu et al., 2004). Under such conditions, principals are seen as both ultimately responsible for school improvement (Shiu et al., 2004; Finnigan, 2010) and for innovating and “triggering change” (Murphy, 2008).

Such rhetoric has been reinforced and expanded by efforts to develop “turnaround^1^” leaders (Peck and Reitzug, 2014), the focus of the program in which this research occurred. Therefore, aligned with the policy rhetoric, it seems likely that the program would emphasize more agentic characteristics and behaviors. This framing may serve to reinforce rather than reduce gender stereotyping related to the role. This research investigates this phenomenon directly, exploring how gender stereotyping influenced the experiences of participants in a turnaround principal preparation program and contributing to our collective understanding of why a gender gap in school leadership exists and closer to how to rectify it.

### Theoretical frame: social role theory

The social role stereotypes of “women take care and men take charge” (p. 1307) impact biased evaluation of women in leadership positions and are both pervasive and resilient (Hoyt and Burnette, 2013). Indeed, despite recognition that more communal traits are often perceived as valuable in leadership roles (Eagly and Carli, 2003), perceptions regarding the required characteristics for successful leadership continue to be strongly linked to agentic or masculine traits (Heilman, 2012). Further, women remain disadvantaged in leadership roles, and more so in leadership positions typically dominated by men (Eagly, 2007). More troubling, implicit bias arises when individuals are unaware of holding biased or prejudiced feelings toward a particular group (e.g., women in leadership). This bias occurs automatically or unconsciously and has formed over years of environmental influences (Kawakami and Dovidio, 2001; Rudman, 2004). In practice, as illustrated in work by Koenig and Eagly (2014) such biases can then produce stereotypes regarding which genders “belong” to which positions within an industry and in the case of this research, a school (e.g., woman as teacher, man as principal) can impact behavior including ease of entry to a given field.

At the same time leadership is defined in these masculine terms, prescribed social roles for women are often framed as communal (e.g., nurturing, caring, demur). Therefore, when women then exercise leadership as traditionally defined (i.e., agentic) they often experience a backlash for violation of their prescribed social roles (e.g., Heilman and Okimoto, 2007; Rudman et al., 2012). This includes being more negatively evaluated when compared to men adopting such behaviors (Eagly et al., 1992). Alternatively, if women deploy more communal modes of leadership and stay true to biased gender expectations, they risk being seen as ineffectual leaders. In this way, women are placed in a kind of double bind in which, regardless of their leadership style, they are likely to encounter great resistance (Koenig et al., 2011). As already highlighted, such resistance may appear as biased negative performance evaluations from external sources, loss of opportunity for leadership positions, and disparate treatment in the workplace (Heilman, 2012; Rudman et al., 2012).

Researchers have identified such barriers as being connected to what can be understood as second generation bias—“powerful but subtle and often invisible barriers for women that arise from cultural assumptions, organizational structures, practices, and patterns of interaction that inadvertently benefit men while putting women at a disadvantage” (Ibarra et al., 2013, p. 60). Second generation bias stems from both implicit bias and perceptions of lack of fit based on gender roles, which negatively impact women. Second generation bias is difficult to recognize and more difficult to counteract (Ely et al., 2011) and can detrimental to women as they attempt to navigate areas that are traditionally held by men, including in this context, educational leadership positions. Moreover, though largely absent from the literature, these same biases may impact the messages received in preparation or other training programs meant to grant participants access to such leadership positions (i.e., principal preparation programs).

We sought to address this gap by utilizing an in-depth comparative case study method examining the experiences of two participants (female and male) in a turnaround principal preparation program. Using social role theory, the following research questions were used to guide our study:

RQ1: How did gender impact the participants' understanding of him/her self as leader prior to and during the leadership development program?

RQ2: How did gender impact participants' experiences (e.g., feedback, opportunities) in a leadership development program (i.e., principal preparation program)?

## Method

This data comes from a larger study that examined how participants in a turnaround principal preparation program came to understand and, later, exercise their roles. In prior work utilizing this data we looked across the sample to analyze emerging themes regarding male and females differing experiences in the program and resulting understandings of leadership and themselves as leaders (Weiner and Burton, unpublished paper).

For this paper, we shift our focus to more closely examine the experiences of two participants in the program to gain a deeper and more holistic account of a phenomenon (Yin, 2009). To achieve this, we took a comparative case study approach (Yin) as it allowed for an examination of divergent experiences thus aligning with our goal to explore gender differences within the context of social role theory. This method was particularly appropriate for this investigation given our focus on examining the experiences of men and women in principal preparation programs. We recognize that gender is one of multiple identities held by our participants (e.g., race, ethnicity, sexual identity), however, as noted by Reed (2012), when race is considered, gender bias remains a significant issue for women in positions of leadership. Further, we note a need for a deeper investigation of issues of gender in leadership development, as female participants in our previous work held various backgrounds and identities yet experienced bias based on gender (Weiner and Burton, unpublished paper).

In particular, this method facilitated our ability to contrast these participants' experiences to help reveal more nuanced patterns that might otherwise be hard to detect in the feedback and support provided to each participant individually (Miles and Huberman, 1994). The comparative case study method has been used within contexts, including education, that have focused on the individual (e.g., teacher) as the unit of analysis (Pascual et al., 2011) and hence was deemed both useful and appropriate for our analytic goals.

### Procedure

This study was carried out in accordance with the recommendations of The University of Connecticut institutional review board (UConn IRB) guidelines. The protocol was approved by the UConn IRB and all participants signed consent forms regarding participation. Each participant was interviewed 4 times over the year at 3 month intervals, each interview lasting approximately 1 h. Initial interviews were structured focusing on the participants' professional background, motivations for engaging in the program, views on principal leadership and what they hope to gain from the program. Later interviews asked participants to reflect and consider whether and how their views had evolved over time (e.g., “In what ways have your views of *leadership* changed as a result of your program experiences? In what ways have your views of *yourself as a leader* changed as a result of these experiences?”).

As our exploration of role identity emerged over time, early interviews did not ask questions related to this issue directly. In contrast, later included questions responsive to participants' emerging understandings and experiences and hence spoke more directly to issues of gender and how they played out within the context of the program. All interviews were digitally recorded and transcribed verbatim.

### Study site

This study took place in mid-size state that is currently running a principal leadership preparation program designed to produce “turnaround leaders.” The program included a month long summer intensive, an apprenticeship as a “resident” in a school, on-going coaching in the placement, as well as monthly seminars. Participants (*n* = 9) receive their principal licensure as a result of successfully completing the program and are supported in finding placement as a principal in a low-performing school in the state. We selected two members of the cohort, a man and a woman, as subjects for this study (see Table 1 below). Our decision to focus on these individuals was two-fold. First, the subjects were most similar in their professional and demographic backgrounds. Though the participants were different in their gender and race, both were in their late 30s with young children at home and both had been teacher leaders in their respective schools prior to entering the preparation program. They were also considered to be leaders in the cohort and scored very well on all program assessments. Second, we believed that their stories were particularly illustrative both in terms of representing the larger experiences of the men and women in the cohort, regardless of other identity features, and regarding the intersection between social and gender identity in the preparation program and in their understanding of and engagement as a school leader.

**Table 1:** **Participant demographic/professional information**.

	**Race**	**Gender**	**Age**	**Sexual identity**	**Marital status**	**Education**	**Past experience**
Tom	White	Male	Late 30s	Heterosexual	Married	Master's degree	Elementary Teacher, Teacher leader
Thali	Non-white	Female	Late 30s	Heterosexual	Married	Master's degree	Elementary Teacher, Teacher leader

### Analysis

Our analysis procedures were informed by our theoretical framework, which is the method preferred in analyzing case study data (Yin, 2009). We analyzed the data thematically using a deductive coding scheme (Boyatzis, 1998). Deductive codes were derived both from current research on the factors that may influence women to refrain from taking on principal roles (e.g., lack of family support) or struggle once in the position (e.g., negative feedback from colleagues) (see Eckman, 2004, for a review) and social role theory literature (Eagly and Wood, 2012). Specifically, Eagly and Karau's (2002) conceptualization of role congruity served to frame understandings of participants' descriptions of leadership, as agentic or communal. In addition, whether the feedback provided to participants was framed as agentic or communal. We also allowed inductive codes to emerge. For each time point, we first independently coded both the interviews and then discussed our codes and emerging understandings from the data until agreements were met regarding interpretation and future coding procedures. This process produced a number of additional codes and facilitated inter-rater reliability. As an example, we found that participants told an “origin” story regarding their reasons for pursuing leadership and how these experiences impacted their current orientation toward leadership (e.g., as a struggle, as a natural progression). We repeated this deductive/inductive process for each time segment as we refined our codes. We returned frequently to the data to ensure coherency and a grounding in participants' experiences. The table below (Table 2) provides the most salient themes that emerged from our analysis and includes each code and representative quotes in support of that code.

**Table 2:** **Table of codes**.

**Code**		
Individual leadership narratives	Leadership as a natural progression	Leadership as struggle
	They [the teachers] had been teaching for, you know, anywhere from 10 to 20 years, a lot of experience, a lot of leadership positions within the school; being on committees and other things like that. And they took me under their wing, and really—really helped me grow, really understand the curriculum, understand the school, just their experience imparting on me (Tom)	Every single principal has told me that I am crazy for going into this program, every single one, which, including my husband who says, “Do you see principals are dropping left and right? No one can even keep them and why are you going into something that everyone is telling you not to?” (Thali)
Leadership development feedback	Build on your strengths	Tone it down
	That's one that is so important as a principal…I think I have a long way to go, but I feel with experience, having those conversations and kind of maybe taking your lumps on some of them and maybe winning some of the battles a little bit as a communicator. I feel like I am getting there for that, but I am really looking forward to this year to help. (Tom)	Because I tend to come across very passionate on education which sometimes could be taken wrong because sometimes the passion may come across as being aggressive or narrow-minded, or that was some of the feedback that I had gotten. (Thali)
Leadership selection	Validation of fit (Hired)	Rejection of fit (Passed over)
	And I think that's why I got this job, because I really had that message—that high expectations for what we're doing, and for teachers, and for kids. (Tom)	He's awesome—trust me. Like, I am not saying he's not, but when I start to look at who, you know, who am I competing against, I'm thinking her and I. And then this happens—he gets a callback and she doesn't even get a callback—and I'm going…I can't shake the—and then to get the verification of they were really looking to a male leader. (Thali)

### Findings

#### Leadership narratives: following the tide or swimming against the current

Initially, Tom and Thali framed effective leadership as communal. Both participants emphasized the need for the principal to be out among the teachers, “to be the lead teacher, to lead by example” (Thali), and accessible to the students, for example by “greeting everyone” each day in front of the school (Tom). The goals of these activities being to build a positive school culture based on trust and caring relationships between teachers and students. Yet, despite these similarities regarding their descriptions of how an effective principal should behave, Thali and Tom described themselves as leaders and their journey to becoming enrolled in the principal preparation program quite differently. Thali framed herself as a fighter and her journey as an ongoing and uphill battle, while Tom framed himself as a born leader and his journey to the program as a natural progression filled with positive encouragement and reinforcement.

For example, when asked about how he ended up enrolling in the principal preparation program, Tom emphasized that he considered himself a “natural leader” having been drawn to positions of leadership from an early age. He explained, “I just loved working with people to try to solve problems, to try to move forward, communicating with people, getting people to gain your trust, leading by example, doing things—showing people that you can do things a certain way, communicating; having honest conversations with people.” Here and elsewhere Tom presented leadership as an extension of himself and personality traits (i.e., he is a problem-solver, he is good with people). In this way, his enactment of leadership becomes just one more part of his identity—to not act as a leader would be more unnatural than taking on such roles.

In Tom's retelling, his leadership capabilities and effectiveness were often reinforced by his colleagues in formal and informal ways. First, Tom recalled that his principal and other administrators often asked him to take on formal leadership roles within his school (e.g., serving on the school leadership team, leading the school discipline team). Second, Tom's colleagues also frequently asked him to represent their interests and guide the school's academic reform efforts. Additionally, when talking about his path to the program, Tom was quick to point out the many ways his colleagues supported his ascension toward leadership. This included some of his earliest experiences as a teacher including joining the fourth grade teacher team. He recalled the impact they had on his experience.

They [the teachers] had been teaching for, you know, anywhere from 10 to 20 years, a lot of experience, a lot of leadership positions within the school; being on committees and other things like that. And they took me under their wing, and really—really helped me grow, really understand the curriculum, understand the school, just their experience imparting on me.

Beyond fellow teachers, Tom also pointed to former and current administrators as a source of encouragement as he took on greater leadership roles and responsibilities. His former principal and district leaders had often connected him with additional opportunities for leadership across the system. They also served as an important support system for Tom's decision-making regarding his future and career. For example, he spoke at length about how his principal and vice principal had helped him decide whether he should enroll in the turnaround preparation program.

We talked about you know the difference between finishing the program at [another organization] and then maybe becoming an assistant principal or a principal that way verse going through this principalship; going through the [Program name] and the—you know we just thought that—they thought and they both did the more of the traditional way through school. But they both thought that this training…was so strong that you know you are going to be fine.

The result of this feedback was a further bolstering of Tom's efficacy and his decision to become a principal. Taken together it seems that, from his earliest recollections, Tom received ongoing reinforcement that he was a leader and should pursue further opportunities to lead.

As much as Tom's story of leadership was framed as natural and smooth, Thali's was framed as arduous. When asked to share how she had come to enroll in the program, Thali began her story in high school and how, as a first generation American of non-college educated parents, she had failed to realize that, as late as her sophomore year, that she was placed in a non-college track. This realization came only when a counselor told her that her dreams for college were unrealistic and that she should consider beauty school after graduation. As she told the interviewers, her story might have ended there but for how her favorite teacher reacted when he heard about the counselor's conversation with Thali.

He marched me right down, swore at the guidance counselor, which at the time when you are in high school you think it's super cool that, like, teachers are swearing at each other…and he was like you know, “You blah-blah-blah! Do your job! This is a good kid. How dare you make choices for her future!” Anyhoo, next day all in college prep classes, completely switched my schedule around.

Thali did end up going to college, with a full scholarship, and saw these early experiences as driving her toward leadership and ongoing advocacy for public school students, particularly those living in poverty and underserved by the system. As she explained,

I have very little tolerance for people that don't believe in our urban kids, because I was there. So it's very hard to have a conversation with me about those things, because when someone is like, “Well, what do you expect from a kid that blah-blah-blah?,” you know I am always like, “Well, that was me. And they are like, “Oh well you are the exception.” I am like, “Well, I shouldn't be thought, see but I shouldn't be the exception; I shouldn't be.”

Throughout her interviews, Thali returned to this incident and how her experiences of being largely overlooked by her teachers drove her desire to be a turnaround principal and an advocate for students she felt were also often overlooked. In this way, her vision of leadership was framed as a mechanism to go against the tide and fight for what she believed.

Like Tom, once she became a teacher, Thali received a great deal of positive reinforcement regarded her effectiveness, winning prestigious national and district awards, serving on a variety of committees and being recruited by her principal to move to with him to a new school to be the reading specialist. That said, unlike Tom, Thali described the enactment of some her leadership roles as fraught, with colleagues often showing active resistance toward her and her efforts. Recalling her work as a reading specialist, she said,

Year one…I cried every day; came home. I mean teachers were like horrible. Like they were just like, “Screw you, and who do you think you are? You are like 12!” You know how that works. I mean you are—when you are young coming in and I remember going home like “Why am I doing this?” And like I kept telling myself, “because that school needs help and they don't see that now.” And I am telling you—it was like—I get goose bumps because like my last year like teachers were like, “I can't believe you're leaving.” And I'm like you hated me! You hated me so much! [Laughter].

Again, though she was inevitably successful in her efforts, it was clear from this story and others that Thali felt that each success was the result of struggle. Moreover, it seems that, as presented by Thali, much of people's discomfort with her leadership had to do with social or cultural norms (e.g., seniority) rather than her skills and knowledge. However, it is interesting to note, that rather than identify these norms as responsible for teachers' response to her, Thali instead tended to identify her way of enacting leadership as the underlying problem. She explained, “I am very annoying…I am like rah-rah-rah! You can do it! Come on guys! And people are like, ‘Oh my God!’ They don't have the energy for that.”

These early experiences of having to rail against negative or discouraging feedback to accomplish her goals was reinforced as she considered whether to apply for principal preparation program. Instead of the support Tom experienced, Thali received a barrage of negative feedback regarding her desire to become a principal.

Every single principal has told me that I am crazy for going into this program, every single one, which, including my husband who says, “Do you see principals are dropping left and right? No one can even keep them and why are you going into something that everyone is telling you not to?” And I am talking about the best of the best and the worst of the worst have said to me, “Trust me, it's bad; don't do it. Just go back into the room and like just live in your four walls. It's really, really bad out there.” But that's what drives me. See, it's just how crazy I am.

Here Thali makes clear that despite her success in her prior roles, few people encouraged her to become a principal. Moreover, this feedback seemed to be oriented toward Thali's capacity, or lack thereof, to deal with adversity (i.e., she will not be able to handle how bad it is). While these negative messages appeared to have done little to deter Thali from her goals, given these warnings from those she looked to for encouragement, one wonders whether, if she were to face difficulties during the program or after, she would have access to supports seemingly so freely accessible to Tom. Additionally, while Thali's narrative did seem to empower her to move forward, by using words like “crazy” and, in other instances, “insane,” to describe her way of engaging in leadership, it positioned her as a kind of “other”—someone who lives outside the boundaries of expectations. Taken together then, we can understand Thali's and Tom's narratives regarding their leadership journeys and themselves as leaders were quite different at the onset of the program and, as we discuss in more detail throughout the paper, seemed to reflect larger gendered messages regarding leadership.

#### Building leadership skills: differentiated messages

We now shift from examining how Thali and Tom presented their leadership journey and themselves as leaders to the preparation program itself. In particular, we focus on how each participant experienced the programmatic feedback and the messages it sent about effective leadership in turnaround schools. To do so, we begin with Tom and Thali's early assessments of their leadership skills and what they hoped to achieve as a result of program participation.

Given the differences in how Thali and Tom framed themselves as leaders, it is perhaps not a surprise that they had different views of what they might gain from the program. First, and in keeping with her prior difficulties with colleagues resistant to her leadership efforts, Thali hoped that the program would help her to “shut up…and just be quiet and listen” and become, “a better listener without already thinking of a rebuttal.” According to Thali, efforts to decrease her “roar” had already produced positive results in her former role.

I have become more polished—way more polished and I was a spitfire, like second, third year of teaching. I mean, I was that teacher that was like, “Ah, no! this is absolutely not right for our kids! This is an injustice!” Where I've learned now that it's like the roar doesn't help sometimes because it actually, makes people shut off, where you have to be very—think about how it is that you are presenting yourself first and then you can kind of, like, lay it out.

Whether warranted or not, Thali framed her goals as focused on toning down her presentation of self and making her views and beliefs more palatable to others. Additionally, by framing this more dispassionate leadership orientation as “polished,” she privileges a measured form of leadership above her own orientation (i.e., rougher). In this way, Thali starts the program naming her style as problematic and in need of reform.

In contrast, Tom framed his desired development in terms of building upon his existing skills. For Tom, this included becoming more forceful in his approach, to “work on the communicating, having the honest conversations with people” so that he could “win” those conversations.

I've improved a lot in the last year with having conversations and communicating with people both bad and good and tough ones. So that's a tough one. That's one that is so important as a principal…I think I have a long way to go, but I feel with experience, having those conversations and kind of maybe taking your lumps on some of them and maybe winning some of the battles a little bit as a communicator. I feel like I am getting there for that, but I am really looking forward to this year to help.

Like Thali, much of Tom's emphasis in building his skills was outwards. He hoped to improve his communication to improve the likelihood that others would listen to and accept his views. However, unlike Thali, who focused on toning down her approach, Tom emphasized that, to communicate more effectively, he needed to be more forceful or aggressive, likening communication to a battle. Additionally, Tom framed his learning as coming primarily with experience rather than through negative reinforcement. Overall, his comments suggest that he felt that while needed development, he was moving toward success (e.g. “I am getting there”), an orientation that fit with his larger narrative of leadership as a natural progression.

Throughout their program participation, these leadership self-assessments were reinforced (i.e., Tom needing to be more forceful, Thali needing to be less so) in a variety of implicit and explicit ways. For Thali, some of this reinforcement came as early as the program's interview process in which candidates participated in a small group interview to collaboratively complete a mock leadership task. During this process, Thali described feeling conflicted by her desire to be assertive in the discussion while also trying to be deferential to other participants' comments. This internal conflict left Thali feeling as though she had not demonstrated her strengths as a leader or an ability to be successful in the program.

And so the reason I say I found it odd was because you are obviously vying for this position, but you also want to be respectful of other's having an opinion. But as you are sitting there, you are going “Now wait, are they looking for someone that is going to be very assertive in this environment?” because obviously, as a leader, you must. Or do you not want to overpower someone because that's also not being mindful of their opinions?…I felt as though I couldn't come across the way I wanted to because I didn't want to overtake the interview, but then in my—and then in my mind, I was like, “Or should I?” I mean, so it was—I almost felt like I couldn't really ah—ah think to the task.

While Thali revealed this internal conflict, Tom only said that he “felt really good leaving that first interview.” He did not express any discomfort or a need to balance or regulate parts of himself to ensure acceptability to others. Rather, he said that the experience was so positive that it convinced him to enroll. As he put it, “What sold it for me was that first interview. I went into that interview going, ‘this is going to be great experience,’ and I left that interview saying, ‘I want this…” While this dichotomy between Tom and Thali could be, in part, attributable to personality differences, given the participants later responses highlighting the often gendered nature of feedback and framing of leadership within the program, it suggest that their feelings may also be partially due to implicit messages about what kinds of leadership were, and would be valued, in the program.

As time went on, these trends in Thali's and Tom's experiences continued and were strengthened by the feedback they received in their program sessions. This feedback was frequent, generally oral and provided to participants in seminar sessions in front of peers. As recalled by Tom and Thali, while some of the feedback focused on content knowledge (e.g., budgeting, human resources, emergency policies), much more pertained to the “softer” skills associated with leadership (e.g., communication, vision, strategy). Moreover it was related to this second group of topics, discussing how leaders should behave in relation to others, that the feedback was understood quite differently based on the participant's gender.

For example, much of the feedback Thali said she received was focused on limiting her emotion and presenting as “less passionate.” She was also told to limit the movement of her hands when she talked and to wear less jewelry because it was “distracting,” and could result in others not taking her seriously. Alternatively, Tom revealed that the feedback he received was to push to “put himself out there,” to “trust his instincts” and exert greater authority in his interpersonal behaviors. He did not mention receiving feedback regarding his appearance or how it might impact others.

Indeed, throughout his interviews, Tom rarely presented program feedback as anything but positive and orientated toward strengthening his already burgeoning leadership capabilities. This was true even when the feedback he received aimed to change Tom's behavior. For example, when Tom asked one of the program facilitators how he might improve his communication to engage more productively in seminar discussions, he recalled receiving the following response,

She [the program facilitator] said that's something a leader would do, so right there I felt pretty good, with some confidence. And then building me back up, and then asking questions, she said, would be a great way to enter these conversations, and “if you don't know, you don't know, but you want to learn, and put yourself out there.” So, I have no problem doing that. So, the next day it all changed for me in a positive way, because of that feedback I got, and I was asking questions, getting involved in the conversation. I felt like I was moving the conversation. And then receiving feedback on that later that day that she was happy that I was using the feedback given the day before, and then a week later reflecting on all of it with the facilitators.

Here, the feedback is presented as both solicited by Tom and targeted toward increasing or enhancing his ability to exert his voice in the conversation. It also seems worth noting that Tom appeared to take this feedback in stride, trying it out almost immediately and with great success. As presented then, it seems that both the nature and the substance of the feedback was, to some degree, aligned with Tom's view of himself as a leader and how he might become more effective.

As the year progressed, Tom continued to receive feedback aimed at helping him to speak up and out and, as he put it, to use more “edge” when communicating with others. According to Tom, the program facilitators told him that this edginess was necessary to lead in turnaround environments where he was likely to encounter resistance from his staff. To support Tom and the other participants in dealing with such situations, the facilitators often used role plays in class, asking the participants to engage in difficult and realistic scenarios to practice their communication skills. When asked to explain what the facilitators meant when they suggested that he build his edge, Tom recalled one of these role plays in which he was meant to address a recalcitrant teacher unwilling to implement the school reform efforts he, as the principal, had laid out. As he explained to the interviewer, this was an example of his edge in that he told the teacher, “We're doing this, and it's going to help kids; it's not just about your kids…That's what we're going to do here. I'm not just going to worry about one class. We're got to worry about the whole school.”

In response to Tom's efforts, one of the program leaders turned to him, applauded his efforts and said, “Tom, you've grown so butch!” Laughing as he told this story, Tom understood the facilitator to be reinforcing his behavior as appropriate and strong. And yet, looking a bit closer, we might also understand this feedback to be fairly autocratic in nature, suggesting that the teacher's views lacked value and that it would be inappropriate for Tom to try to defuse the situation rather than to fight her on the issue. When Tom recalled this and other instances in which he was told to be more aggressive, he made clear that the pros and cons of such an approach were not discussed. The message he received was that an aggressive stance was appropriate when others were challenging his position regardless of the specific context of the situation or the players.

While Tom was being told to act more aggressively, Thali was receiving the opposite feedback—that she needed to tone down her way of communicating. Throughout her interviews Thali mentioned receiving feedback that her communication style was likely hindering her ability to be an effective leader. As she explained,

Right at the end of summer intensive, we had to write where we felt our strengths were, but then also where we felt that we needed improvement on and we had to take that and kind of use that within our residency. And mine was communication and the way that I come across, because I tend to come across very passionate on education which sometimes could be taken wrong because sometimes the passion may come across as being aggressive or narrow-minded, or that was some of the feedback that I had gotten.

Thali, like Tom, presented herself as already aware of her areas for growth and desirous of enhancing her practice. That said, her needs are framed as deficits and not as existing strengths that require bolstering. As presented here, her current way of communicating is wrong in that those experiencing it are made to feel uncomfortable or put off. Additionally, the sources of this ineffective communication is inherent to her personality, it comes directly from her “passion.” This framing seems a sharp juxtaposition to Tom's experience in which the feedback appeared to call for him to communicate greater passion with what might be considered little consideration for whom he put off or made to feel uncomfortable.

Beyond the substance of the feedback, also unlike Tom, Thali gave few examples in which she received positive, reinforcing feedback from program facilitators. Instead, when talking about her areas of improvement, Thali tended to look inwards for affirmation. She explained,

I'm definitely trying to work on putting myself in the lens of others and really listening and analyzing the situation as opposed to automatically coming back with a rebuttal or I can solve that or let me give you my side of it…I'm glad to say that I've been able to, kind of like, really work on it, find my defaults and understand how my body language kind of will change.

Again, through these comments, Thali makes clear that, to be successful, she needs to fundamentally shift her way of being, she must find and reprogram her “defaults.” Additionally, unlike Tom who appeared to receive frequent external reinforcement for his incorporation of feedback, Thali's moves to improve appear to have remained outside the public eye. In these ways, we can understand Thali's experiences to be primarily about suppression and silencing and Tom's to be about activation and voice.

#### No work for the wicked (women)

Despite her reported willingness to change her behaviors to align with program's and perhaps external expectations, upon graduating Thali had substantial difficulty finding a principal position. Tom, on the other hand, was the first in the cohort to be hired as a principal, receiving the news before the end of the program. In reflecting on why this was the case, Tom made clear that part of it was his ability to clearly communicate the strength of his vision, “And I think that's why I got this job, because I really had that message—that high expectations for what we're doing, and for teachers, and for kids.” Later in the interview, Tom went further and directly attributed this strength to the program and how the feedback he received had given him the confidence to present that strong vision.

I always felt like I had the work ethic, and that was never an issue, and they [the program facilitators] challenged that, too, but just the–like the confidence, I felt like the only thing that would be slowing me down would be myself, like I'm getting my own way sometimes with things, just because I might doubt it, or might have confidence about issues about it, and I still a little have those things, but I feel that—I really feel rock solid on why I'm doing this, and it's for the kids.

Overall then, it seemed that the hiring process was fairly smooth for Tom, reinforcing his approach to leadership as well as his prior experiences of leadership as a natural result of continued effort.

In contrast to Tom, not only did it take a long time for Thali to find a job, but she also found the process to be quite painful and emotionally draining. First, despite what she perceived to be her hard work toning down her communication style to make it more palatable to others, the feedback she received during and after interviews was that the school's hiring committee found her to be too aggressive or intimidating. Others said they worried about her ability to discipline students and lead the staff (in comparison to a male candidate). In an attempt to respond to these mixed messages (i.e., that she was both too aggressive and too weak to be a principal), Thali revealed that she had been working harder to “play the game,” doing whatever she could to remove people's negative perceptions about her potential fit or capability to lead. As she explained, in one of her more recent interviews,

I completely played. I did. It was a part of me that I said to myself, “I'm going to speak as though my entire faculty is amazing, and everyone's there for the right reason.” And that's how I spoke. So I spoke in the words of collaboration, shared leadership—which are things I believe in—I can't do it alone…So I know for a fact that I was very soft. I was very cognizant of coming across as overpowering So, I'm telling you, I know for a fact that I mentally told myself, “You have to get in the door.” Right? So this is the way I looked at it. I said, “You have to get in the door and whatever you do when you walk in the door is different than what your interview sounds like.”

However, despite these efforts, Thali was not offered the job. Interested in trying to understand how this strategy of giving the committee more of what she perceived they wanted failed to work, she activated her networks to find out why she was not selected.

I sit there and I'm like, “I am really confused about all of this.” [I] do some investigative work. But the feedback I get from specific people that—not from the interview panel but from other people—was that they were really looking for a male leader for the building. They felt more comfortable being led by a male.

In response to these ongoing disappointing and what Thali was beginning to recognize as gender biased results, Thali initially said that they were making her stronger and more resilient. She continued to express confidence in her skills and her talents and believed her work and effort would lead to a principal position. Given her concerns about being perceived as aggressive and her acknowledgement that her male colleagues were more successful in landing principal positions, Thali attributed her confidence to being more “male,” giving voice to the overconfidence that she witnessed in other male participants in the program and in other aspects of leadership.

And I'm saying—there's nothing else that I would be able to bring to the table. So I'm proud that I've not let this consume, or let it question, my begin—so, I'm proud about that. And, like I said, my learning from it is, I'm super resilient. I am super positive. I know who I am. I know what I can offer. And I don't know if that makes me a male [laughs].

However, whether only for show or deeply felt, as Thali talked further, this resilience began to wane as she made further and deeper connections between her gender and the lack of success she was experiencing on the job market.

I'm really fixated, like, on the male and female thing. I am so—like, I'm trying not to think of it so much, but I can't help but to say to myself—we have, in our cohort…we have six females and three males. [name of colleague], who is…her expertise is, phenomenal. Like, she is amazing. She didn't get a call back. One of our male cohorts did…He's awesome—trust me. Like, I am not saying he's not, but when I start to look at who, you know, who am I competing against, I'm thinking her and I. And then this happens—he gets a callback and she doesn't even get a callback—and I'm going…I can't shake the—and then to get the verification of they were really looking to a male leader. And I am going, “Why? Why do we do this? Like, why do females do this to other females?…That's why I say, like this whole week, or last two weeks, I'm like, I need to really sit and meditate and think about some of this stuff, because what does that mean?

Deconstructing these comments a bit, it seems that part of what is so disappointing to Thali is the lack of control she and her female colleagues have in the hiring process. They can be skilled—even “phenomenal” and will be passed up because of underlying gender issues. Additionally, she seems particularly perturbed about the fact that these hiring committee are often made up primarily of women, as most teachers as women. In this way, Thali situates the problem primarily as one of female-on-female aggression. Unfortunately, by doing so, one might argue that Thali's comments serve to do what she is complaining about (i.e., making women, including herself, the problem) and suggests that the underlying larger issue of social and institutional sexism (i.e., second-generation bias) remained somewhat obfuscated.

Together, her negative experiences on the job market coupled with her confusion and disappointment regarding their cause, left her discouraged and wondering whether it would be possible for her to both hang on to her values and to herself—while remaining in the profession. She articulated this struggle in this way.

It's a bad place to be in, mentally. I don't know what to do with that information, because I won't—you know, someone's like, “Well, you should start your own school.” I won't do that. That's not the solution….Could I do it? Sure. Is it enticing? Sure. Is that how I feel we're going to change the state of education? Nope. I would just create more reasons to why we need to do an us versus them, you know?…So many dilemmas, but—you know, you take your small victories and you go with that and, hopefully, I—I still l have faith and hope. I just have to re-examine and re-determine what that is and what that looks like, and we will go from there.

## Discussion

Whether in schools, corporations, or government, women remain underrepresented as leaders (Dworkin et al., 2015). And yet, though the problem is on-going and well-documented, understanding the differential experiences women and men may have as they are trained for and seek leadership positions is only beginning to be explored (Ely et al., 2011). This lack of knowledge is evident in the context of this study—K-12 education—and particularly principal preparation programs. Taking up this issue directly and deeply examining the experiences of two participants (one male and one female) in a principal preparation program aimed at creating “turnaround leaders,” we found that social role conflict and, in particular, gendered messaging from the program played an important role in how participants came to understand effective leadership and the degree they fit within that frame. Though we have not investigated multiple marginalized identities in this work, we recognize that differential experiences between our participants are also likely influenced by their race and/or ethnic differences (e.g., Livingston et al., 2012) as well as other potential differences in identity. However, as noted in previous research, issues of gender bias have significant negative influence on women in educational leadership across different races and ethnicities and, as such, require further examination in evaluation of educational leadership development (Brooks and Jean-Marie, 2007; Reed, 2012).

First, though, it is important to mention that our findings reinforce that stereotypes regarding leadership and how men and women can exercise leadership behaviors develop long before individuals pursue such positions (Eagly and Karau, 2002). That is, gender influenced how our participants understood and positioned themselves as leaders prior to enrollment in this program. As described by our participants, the “construction and practices of leadership” as experienced by Thali and Tom influenced “who may be considered leaders and who may lead' (Coleman and Fitzgerald, 2008, p. 124). In this case, our male and female participants came into the program with very different conceptualizations regarding themselves as leaders and narratives regarding how their ascent to leadership transpired. Though these differences were likely influenced by other features of identity including Thali's status as a non-white, first generation American citizen, the participants' framings also reflected existing research on how males and females “fit” within existing leadership frames (Eagly and Karau, 2002; Coleman and Fitzgerald, 2008; Hoyt and Burnette, 2013).

Specifically, while the male participant framed himself as a natural leader and his journey toward leadership as well supported and somewhat inevitable, the female participant framed her ascension to leadership as arduous requiring that she position herself as a fighter. Our participants' divergent experiences to leadership reflect role stereotypes, as both leadership and masculine gender roles were perceived as congruent roles for the male participant and incongruent for our female participant (Eagly and Karau, 2002). This perception of incongruity was true even in relation to Thali's closet allies (e.g., husband, mentors, friends) who often took a position of questioning her decision to purse a principal position. In this way, our findings mirror that of prior work that suggests that women may not simply be overlooked as potential leaders but actively discouraging for such aspirations (Rudman et al., 2012). The experiences of our participants highlight the challenges women face when seeking leadership positions such that, “gender status rules virtually guarantee men's greater access to power and resources, resulting in a system that rewards men for leadership abilities while punishing comparable women, thereby reinforcing the perceived conflict between a woman's gender and power.” (Rudman et al., 2012, p. 176).

When considering the impact of gender on the participants' experiences in the program, we want to first call attention to the fact that the site selected for this study was unique in that there were a greater proportion of female participants than would typically be found in principal preparation programs. This fact, on its face, would appear to be an initial attempt to increase the pipeline of women into school leadership (Darling-Hammond et al., 2007). Alternatively, we might anticipate that this disproportionality would have served to temper traditional gendered messages regarding situating leadership as a male endeavor. However, our findings revealed that, as a result of the feedback and differential support provided throughout the program based on participants' gender, our participants' understandings of leadership and effective leadership were situated in a gendered frame. That is the program supported a “gender skewed” view of leadership that supported a “good” leader as a male leader (Coleman and Fitzgerald, 2008). This framing left our female participant struggling from initial entry into the program to the exit, but our male participant received encouragement and reinforcement of a gendered (i.e., agentic) approach to leadership.

The findings from our study call attention to the problems of educational leadership development, and in this particular case, principal preparation programs, when such programs fail to identify, name, or address that leadership is conceptualized and understood that reflect the dominant normative culture. Such reflections include, as seen in our study gendered norms and gendered roles as well as other constructions of leadership that favor whiteness (Banks, 2000; Brooks and Jean-Marie, 2007; Reed, 2012), heterosexuality (Tooms, 2008; Muhr and Sullivan, 2013), and likely other, though so far understudied, features of identity. This failure to acknowledge, discuss or challenge these views of leadership, and in this example, reinforce these views on gender, advantaged the male participant while subsequently disadvantaging the female participant. Also, given that the program had enrolled a greater number of women in the program, our findings revealed that the “add women and stir” approach to leadership development programs is an ineffective approach to developing women leaders when there is no active discussion of how leadership is conceptualized and operationalized as gendered. As was evidenced in this study, the female participant in this principal preparation program was negatively impacted by this lack of discussion of the gender stereotyping of leadership and bias women experience when exercising leadership (Coleman and Fitzgerald, 2008; Ely et al., 2011).

### Issue of identity as a leader in development program

Developing an identity as a leader is a critical component to leadership development. Failure to include discussion of identity writ large and gender identity in particular in the discussion of leadership identity development within this program was highly problematic for the female participant in our study. Failure to talk about gender identity and its impact on leadership identity both projected and experienced by Thali was “fraught at the outset” as women in leadership “must establish credibility in a culture that is deeply conflicted about her authority” (Ely et al., 2011, p. 477). As supported in our findings, throughout the process of leadership identity development our female participant received messaging both prior to her arrival in the principal preparation program that questioned her credibility as a leader, and throughout the program as she received messages to “tone down” her behavior (i.e., be less agentic). When she embraced more communal characteristics, based on feedback she received during the program, when interviewing for principal positions, she was told that a male leader was more valued for the position because of the perceived need for a leader demonstrating agentic characteristics by those on the hiring committee.

Together, these experiences suggest that the principal preparation program failed to provide her with the support to understand the contradictions and biases she would face in her leadership role (Coleman and Fitzgerald, 2008), and in fact may have contributed to the contradictions and exacerbated the challenges she faced reinforcing second-generation bias within principal preparation (Ely et al., 2011). As experienced by Thali, the approach taken for leadership development within this study was to teach our female participant to act like a man. However, whether intentional or not this approach was misguided as it failed to provide Thali adequate “strategies for countering the effects of gender bias” encouraging her instead “to become overly focused on self-image” (Ely et al., 2011, p. 488) via her presentation to others.

Given the understanding that leadership development can offer opportunities for transformation of organizations (Harris and Leberman, 2012), evaluation of leadership development programs (principal preparation) is one mechanism available to influence the advancement of women into educational leadership. However, as supported in our findings, these leadership development programs must account for and directly address second-generation bias if such programs are to increase women's representation in educational leadership (Ely et al., 2011). As such, in the section to follow we offer implications to help enhance opportunities for women enrolled in principal preparation programs.

### Limitations

This study is not without limitations. As this is a comparative case study of two participants from a single principal preparation program, it was neither the objective of this study nor would it be appropriate to generalize findings to other principal preparation programs. The research was also focused on the participants' perceptions regarding their program experiences. As such, we did not gather data regarding those interacting with participants nor include observations of those moments. Therefore, additional research is required to better understand how and which programmatic elements serve to shape participants' views. Further, as noted early, our analysis surfaced issues specific to gender, though we recognize that race/ethnicity and other identities impact gender role stereotypes and occupational fit (Hall et al., 2015). In education, this research is still in development (Loder-Jackson, 2009) though it suggests that such identities are important and may create additional barriers to when they intersect (e.g., Reed, 2012). That said, work outside the field of education that has examined the experiences of black women demonstrating agentic leadership behavior found that they received less negative responses to such displays when compared to dominant white female leaders (Livingston et al., 2012). Further black women were comparably positively evaluated with white male and white female leaders when leading successful organizations (Rosette and Livingston, 2012). However, black female leaders can face burdens as a result of the intersection of multiple identities, including race. Therefore, future research must examine how multiple marginalized identities intersect to impact participants of a leadership development program (e.g., Nichols and Nichols, 2014). A final limitation to note, as women in another male-dominated field, we had a personal interest in exploring these issues and how they might be mitigated. To help ensure that our personal goals did not drive our analysis we utilized member checks and feedback on the analysis.

### Implications

We offer the following suggestions for consideration to enhance opportunities for women in leadership development programs, with a focus on educational leadership development programs in particular. First, such programs need to address issues of identity including gender, second-generation bias, and how this bias influences leadership within the context of educational leadership (Coleman and Fitzgerald, 2008; Ely et al., 2011). This includes discussion, at the point of entry into the program, regarding participants' and instructors' current narratives regarding how they frame and understand leadership and themselves as leaders. Discussing and unpacking such narratives has been suggested as a tool to enhance participants' ability to engage in critical reflection (Brown, 2004; Sperandio and LaPier, 2009) and can be considered a critical element of adult learning and development (Brookfield, 1995).

Additionally, principal preparation programs should also include examination, by both participants and instructors, of biases and stereotypes regarding leadership, including discussion of the impact of these biases and stereotypes on women and other minorities when they exercise leadership. Without this examination, instructors and participants can reinforce (implicitly or explicitly) biases and stereotypes of leadership that negatively impact participants in these programs. By recognizing and naming biases and stereotypes, leadership development programs can “give participants a more nuanced understanding of the subtle and pervasive effects of gender bias, how it may be playing out in their development as leaders, and what they can do to counter it” and help make participants less susceptible to the negative outcomes of these challenges (Ely et al., 2011, p. 486). Further, principal preparation programs should focus participants' efforts toward leadership development based on the participants' meanings, values, and purpose for seeking leadership. Remaining grounded to their larger purpose for leadership allows women in particular to stay focused when faced with conflicting messages about how they are to behave as leaders (Ely et al., 2011). Also, researchers must examine how the intersection of multiple marginalized identities impacts participants in leadership development programs. Recent work examining the intersection of gender and race on perceptions of leadership indicate that race and ethnicity has differential impacts on women (Livingston et al., 2012) and those impacts will also have implications for women of color enrolled in educational leadership development programs.

Finally, despite the emotional strain it caused, some might consider the final outcome of Thali's experience as positive; she was eventually hired as a principal in a turnaround school. And yet, we would caution that while parity in employment is important, it is also important to consider the potential different challenges leadership positions, even among similarly categorized organizations leaders of different genders might face. Indeed, research suggests that women are more likely to be selected for precarious, as compared to more favorable, leadership positions (e.g., organization that has undergone a crisis) (Haslam and Ryan, 2008). Naming this phenomenon the “glass cliff,” researchers have found that, in an effort to signal that an organization is attempting to improve performance women may be perceived as a more appropriate leadership choice (Kulich et al., 2015). Therefore, the issue of the “glass cliff” should be examined within the context of educational leadership and specifically in turnaround schools, as the underlying causes driving underperformance and hence resources for improvements may vary greatly across such schools and potentially place women in more precarious leadership positions than their male colleagues.

## Conclusion

This work is significant in that it gives new insights into why women are still underrepresented as principals and perhaps in educational leadership positions more broadly. Further this work begins to address the need to better understand the intersection of gender and leadership development within the context of educational leadership (Coleman and Fitzgerald, 2008). Our findings suggest that women's narratives regarding their understanding of principal leadership and their perceptions of their leadership capabilities may lead to a dissonance not experienced by their male colleagues. While such a narrative may provide women a certain strength and resiliency to fight for their place at the table, it may also make them more vulnerable to internalizing critiques that are gendered but not explicitly stated as such. Our findings also support recommendations that discussion of gender role stereotyping and second-generation bias must be included in educational leadership development programs. Further, such programs must also consider the degree to which current discourses on “turnaround” leadership serve to reinforce existing stereotypes about leadership as a primarily male endeavor within educational leadership. Such inquiries could help to develop new narratives and interventions to support both women and men in leadership development and help to produce a new and more equitable generation of principals.

## Author contributions

Both authors made significant contributions to all phases of manuscript development.

### Conflict of interest statement

The authors declare that the research was conducted in the absence of any commercial or financial relationships that could be construed as a potential conflict of interest. The reviewer, MEB, and handling Editor declared their shared affiliation, and the handling Editor states that the process nevertheless met the standards of a fair and objective review. The reviewer, ACP, and handling Editor declared their shared affiliation, and the handling Editor states that the process nevertheless met the standards of a fair and objective review.

## Footnotes

^1^Meant to “spur systemic reform to improve teaching and learning in America's schools,” (U.S. Department of Education, 2012), “turnaround” includes four school intervention models: (1) *Turnaround:* the school is redesigned and the principal and at least half the staff are replaced. (2) *Restart*: The school is converted to a charter. (3) *Transformation*: The school engages in redesign including replacing the principal. (4) *School closure*: The school is closed. This program trained principals to serve in transformation and turnaround schools.
